# Fabrication of TiN nanostructure as a hydrogen peroxide sensor by oblique angle deposition

**DOI:** 10.1186/1556-276X-9-105

**Published:** 2014-03-04

**Authors:** Zheng Xie, Xiangxuan Liu, Weipeng Wang, Can Liu, Zhengcao Li, Zhengjun Zhang

**Affiliations:** 1 State Key Laboratory of New Ceramics and Fine Processing, School of Materials Science and Engineering, Tsinghua University, Beijing 100084, China; 2 High-Tech Institute of Xi’an, Xi’an, Shannxi 710025, China; 3 Key Laboratory of Advanced Materials (MOE), School of Materials Science and Engineering, Tsinghua University, Beijing 100084, China

**Keywords:** Titanium nitride, Nanostructure, Oblique angle deposition technique, Hydrogen peroxide, Sensor

## Abstract

Nanostructured titanium nitride (TiN) films with varying porosity were prepared by the oblique angle deposition technique (OAD). The porosity of films increases as the deposition angle becomes larger. The film obtained at an incident angle of 85° exhibits the best catalytic activity and sensitivity to hydrogen peroxide (H_2_O_2_). This could be attributed to its largest contact area with the electrolyte. An effective approach is thus proposed to fabricate TiN nanostructure as H_2_O_2_ sensor by OAD.

## Background

Nanostructured electrodes have stimulated great interests due to their potential applications in the areas of online real-time analysis and sensitive detection [1,2]. To meet the demand in those applications, electrodes need to have some important criteria including large specific area, high electrochemical activity, and good biocompatibility. In recent years, nanorod arrays directly grown on a current collector have been investigated as nanostructured electrodes for biosensor application since their well-defined one-dimensional (1D) structure is favorable for electron conducting and ion accessing [3]. Due to the exceptional combination of chemical, physical, mechanical, and electrical properties, titanium nitride (TiN) attracts much attention for their potential application in various fields such as protective coating [4], supercapacitors [5], and catalysis [6,7]. Recent literature has also reported its potential use as electrodes for pH sensor [8] and hydrogen peroxide (H_2_O_2_) sensor [3]. H_2_O_2_ is not only a byproduct of a wide range of biological processes but also an essential mediator in food, pharmaceutical, clinical, industrial, and environment analysis [9]. Therefore, it is of great importance to achieve sensitive and accurate determination of H_2_O_2_. TiN nanorod arrays (NRAs) are expected to possess good conductivity and biocompatibility with unique 1D nanostructure, making a superb electrode for H_2_O_2_ sensor.

The TiN NRAs can be obtained by a great number of methods, such as electrospinning [10] and solvent-thermal synthesis [3]. However, all the aforementioned methods need a nitridation treatment of TiO_2_ nanorods in ammonia atmosphere at a high temperature. Therefore, a facile and one-step fabrication method to prepare TiN NRAs is in demand. Oblique angle deposition (OAD) technique is an electron beam evaporation method, which has been used in the industry for fabricating one- or two-dimensional materials at large-scale production with relatively low cost. It provides a simple way to produce large area, uniformly aligned nanorods with controlled porosity. During the OAD process, the vapor flux is deposited onto a substrate at a large angle *α* with respect to the substrate normal, and a well-aligned and separated nanorod arrays can be obtained due to the self-shadowing effect [11,12], with growth orientation toward the vapor flux direction [13]. Moreover, the porosity can be readily tuned by varying the oblique angle, and various substrates such as glass, F-doped SnO_2_ (FTO), Si, etc., could be deposited on.

In this work, we report a one-step method, i.e., by OAD method using electron beam evaporation for fabricating TiN nanostructure with tunable morphologies and porosities. The TiN nanostructures are used as the electrodes for electrochemical sensing H_2_O_2_, exhibiting good performance.

## Methods

### Fabrication of TiN films by OAD

The TiN NRAs were deposited on silicon and FTO substrates using OAD described elsewhere [14]. The substrates were sequentially cleaned in acetone and alcohol by ultrasonic washer and then rinsed in deionized water for 5 min each. The system was pumped down to a base pressure of 2 × 10^−5^ Pa, and then the TiN films were deposited at a deposition rate of 0.5 nm s^−1^, which was monitored by a quartz crystal microbalance. The deposition angle of TiN flux was set at *ca.* 0°, 60°, 70°, 80°, and 85° with respect to the substrate normal, respectively. The substrate temperature was maintained at *ca.* −20°C with liquid nitrogen.

### Characterizations

The crystal structure of the TiN films was characterized by X-ray diffraction (XRD Rigaku 2500, Shibuya-ku, Japan ), which was conducted from 20° to 60° at a scanning speed of 6° min^−1^, using Cu Kα radiation (*λ* = 0.15406 nm). The morphology was characterized with a field emission scanning electron microscopy (SEM JEOL-7001 F, Akishima-shi, Japan) working at 20 kV. The microstructures of the prepared samples were characterized in detail with a transmission electron microscope (TEM JEOL-2010 F). The refractive index (*n*_e_) of the TiN films deposited at various oblique angels was measured by spectroscopic ellipsometry (J.A. Woollam, Co., Inc., Lincoln, NE, USA).

Electrochemical measurements were carried out in a 250-mL quartz cell connected to an electrochemistry workstation (CHI 660, Shanghai Chenhua Instrument, Shanghai, China). A three-electrode assembly was adopted for the test, with the TiN films as a working electrode, a Pt foil as a counter electrode, a saturated Ag/AgCl as a reference electrode, and phosphate buffer solution (PBS, pH 7.0) as the electrolyte. The current versus time was recorded at −0.2 V bias versus saturated Ag/AgCl.

## Results and discussion

**Figure 1:**
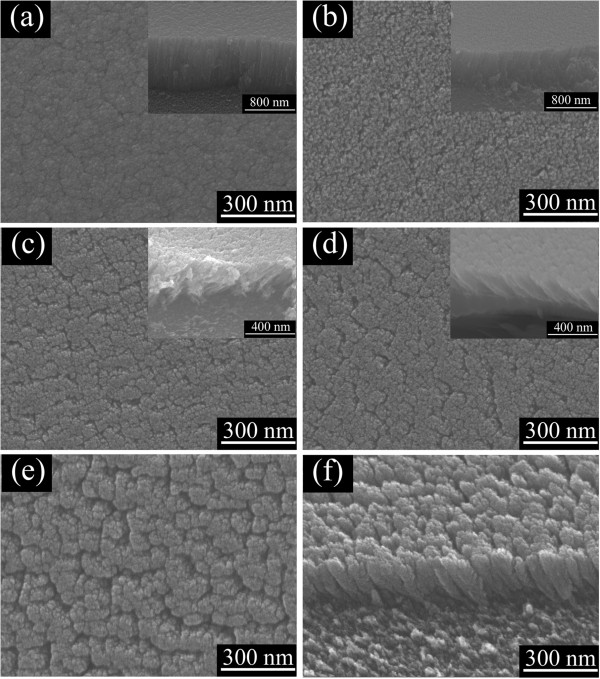
**Top view SEM images of TiN films deposited at various oblique angles. (a)** 0°, **(b)** 60°, **(c)** 70°, **(d)** 80°, **(e)** 85°, and **(f)** side view image of **(e)**. Insets show the side view images.

Figure 1 shows the typical growth morphology of the TiN films deposited at various deposition angles. In the same deposition time of 30 min, the thickness of film gradually decreases from 860 to 190 nm as the deposition angle increases from 0° to 85°. It is because that the incident flux accepted by the substrate within a certain deposition time decreases at larger deposition angle [12]. The film morphology is obviously dependent on the oblique angle. For the film deposited at 0°, i.e., vertically deposited, a dense and flat surface was obtained as shown in Figure 1a. When the deposition angle was ≥60°, porous nanostructure was formed as shown in Figure 1b,c,d,e. It has been illustrated that during the OAD process, self-shadowing effect and limited surface diffusion lead to the formation of distinct columnar structure [11,15]. With the deposition angle further increased to 85°, an aligned self-standing TiN nanorod arrays with length of *ca.* 270 nm and diameter of *ca.* 90 nm was obtained, which can be seen from the side view image in Figure 1f.

**Figure 2:**
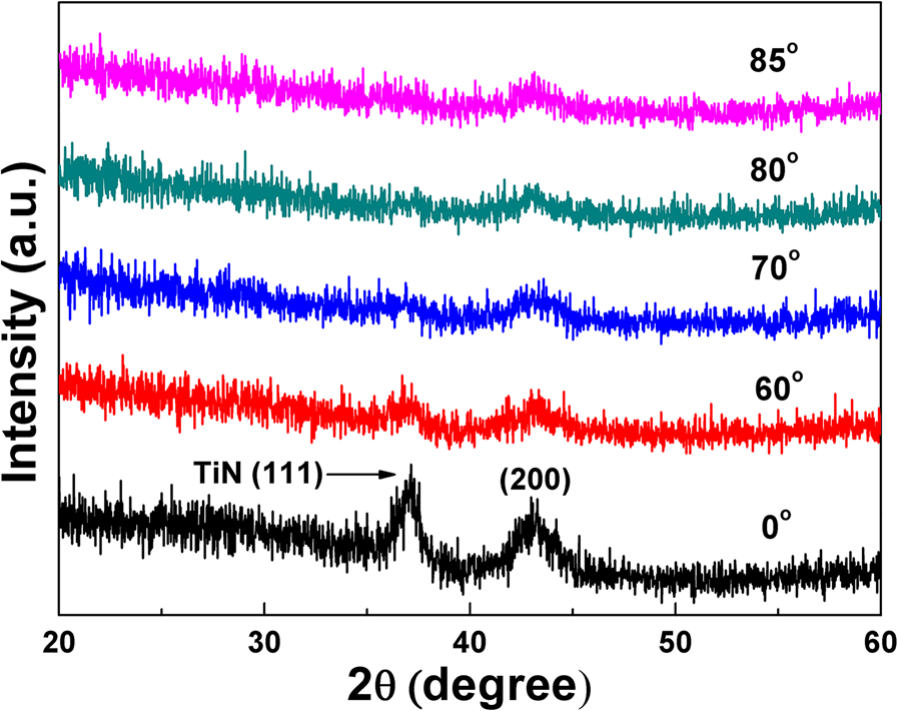
XRD patterns of the TiN film deposited at various incident angles.

Figure 2 displays the XRD patterns of the TiN films deposited at various incident angles. It can be seen that the TiN film deposited at 0° exhibits (111) and (200) diffraction of the face-centered cubic (FCC) structure of TiN (JCPDS 38–1420). The (111) peak becomes weaker for the films deposited at ≥60°, which can be attributed to the decrease in film thickness [16] and the formation of nanostructure during the OAD process.

**Figure 3:**
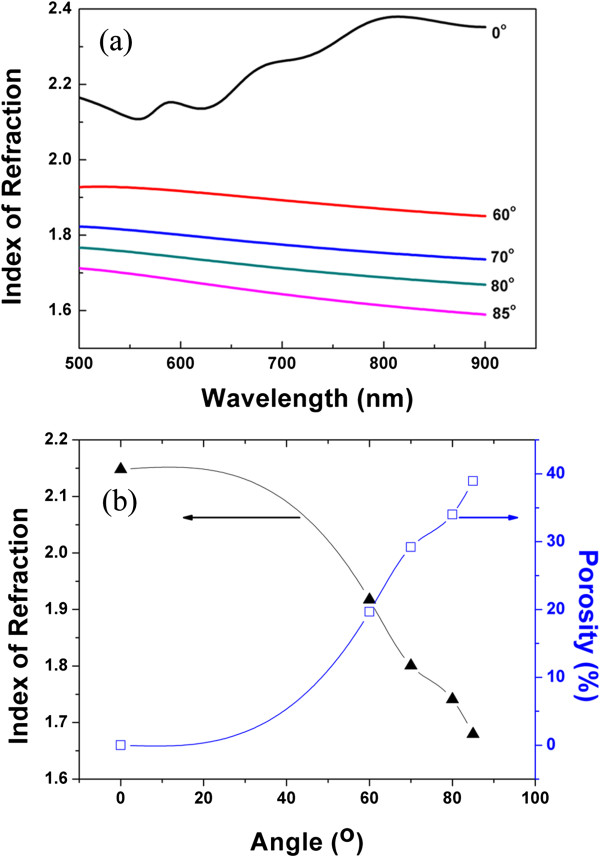
**The refractive index spectra and refractive index at a wavelength of the TiN films. (a)** The refractive index spectra of the TiN films in the wavelength range of 500 to 900 nm. **(b)** The refractive index at a wavelength of 600 nm and the calculated porosity of the films, as a function of the oblique angle.

The refractive index (*n*_e_) of the as-prepared TiN films was measured by spectroscopic ellipsometry at wavelengths from 500 to 900 nm. Figure 3a plots the refractive index of the TiN film as a function of the wavelength. One can see that the film refractive index diminishes with the increase of the deposition angle. For a clear demonstration, we plot the variation of *n*_e_ at 600 nm as a function of the deposition angle, which is illustrated in Figure 3b. As the deposition angle increases from 0° to 85°, *n*_e_ decreases from 2.15 to 1.68, which is the result of the formation of nanostructure [17]. For two non-absorbing components with volume fractions *f*_i_ and refractive indices *n*_i_, the Bruggemann effective medium approximation gives [18]

$$\begin{array}{ll} f_{1} & \left[\left({n_{1}}^{2}-{n_{e}}^{2}\right)/\left({n_{1}}^{2}+2{n_{e}}^{2}\right)\right]\\ & +f_{2}\left[\left({n_{2}}^{2}-{n_{e}}^{2}\right)/\left({n_{2}}^{2}+2{n_{e}}^{2}\right)\right]=0. \end{array}$$

Herein, *n*_e_ of a porous film is given by an average of air and material when the pore size is much smaller than the wavelength. Using the *n*_e_ at 600 nm, the porosity of the above TiN films is calculated using the Bruggemann approximation, and the result is displayed in Figure 3b. When the deposition angle is increased, the porosity increases and reaches the maximum at the deposition angle of 85°, which is in accordance with that observed by SEM (see Figure 1).

**Figure 4:**
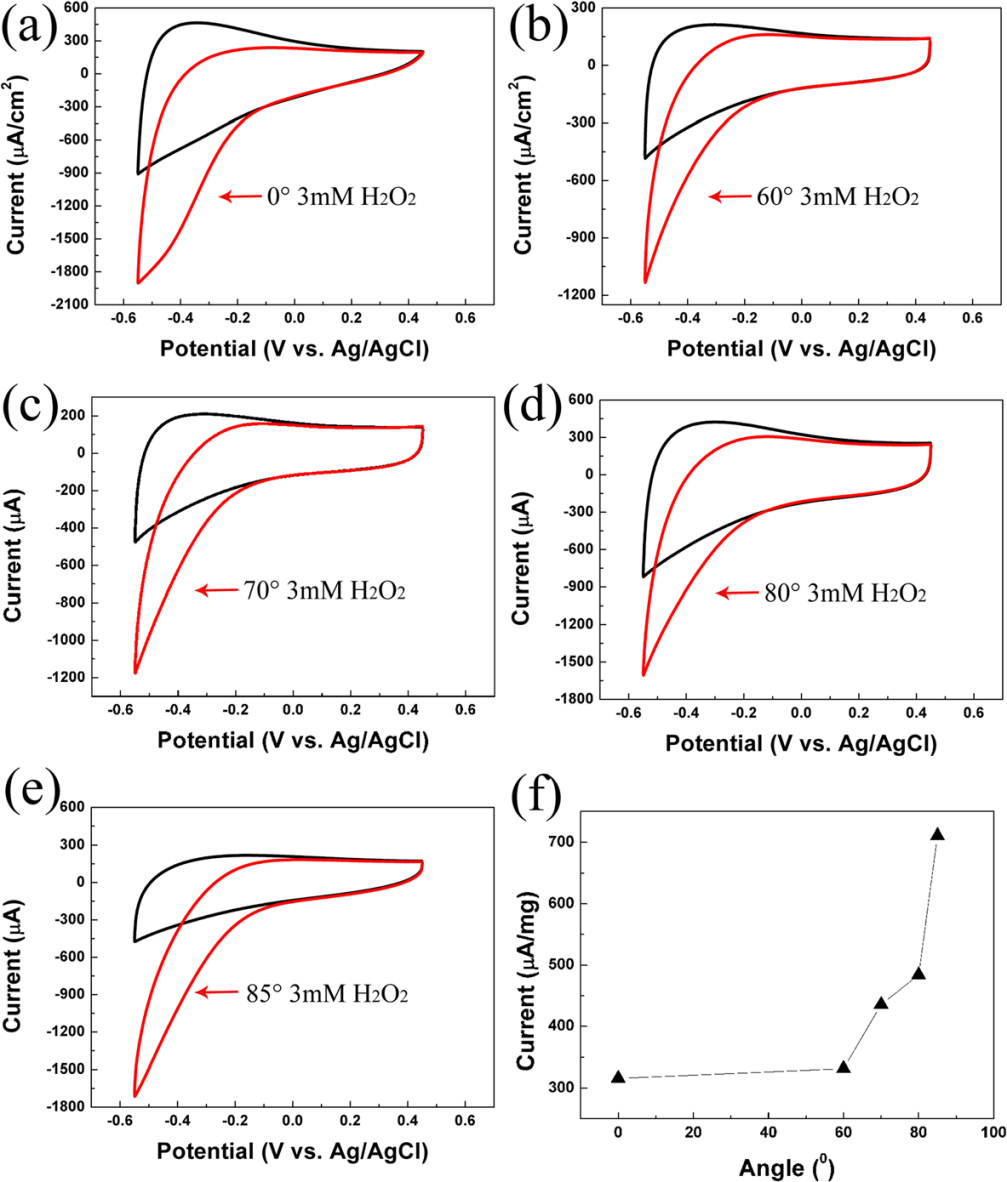
**The C-V curve before and after adding 3 mM H**_**2**_**O**_**2 **_**for TiN films deposited at various angles. (a)** 0°, **(b)** 60°, **(c)** 70°, **(d)** 80°, **(e)** 85°, and **(f)** the relationship of ∆*I* versus deposition angles.

Figure 4a,b,c,d,e presents the cyclic voltammograms (CVs) of these TiN films in the absence and presence of H_2_O_2_ in 0.2 M PBS with pH 7.0. The reduction current increases with the addition of 3 mM H_2_O_2_, indicating an obvious catalytic reduction of H_2_O_2_ on the electrode [3]. Generally, the current difference, Δ*I* [(Δ*I* = *I* (presence of H_2_O_2_) − *I*_0_ (absence of H_2_O_2_)] at −0.2 V is adopted as a key index to evaluate the sensitivity for H_2_O_2_[19], (Δ*I* reflects the sensitivity of detecting H_2_O_2_) Accordingly, Δ*I* is plotted as a function of the deposition angle in Figure 4f, where the Δ*I* in the unit of microampere per milligram has been normalized to the sample weight. It can be seen that Δ*I* increases dramatically with the increase of deposition angle, and the film deposited at 85° shows the best performance, whose current is more than twice as high as that of the film deposited at 0°. The current enhancement is attributed to the significant increase in contact area between the electrode and the electrolyte, which is verified by the aforementioned SEM morphology and porosity estimation.

**Figure 5:**
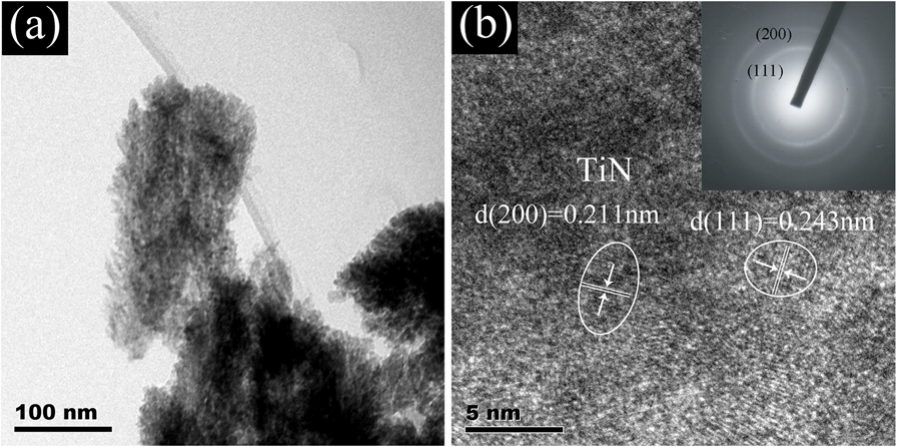
Low-resolution TEM image (a) and high-resolution TEM of the TiN deposited at oblique angle of 85° (b).

In addition, TEM is employed to further study the microstructure of the TiN film deposited at 85°, which is served as a representative sample. From the low-magnification TEM image as shown in Figure 5a, one can see that the nanorod structure is clearly observed with length of *ca.* 280 nm and diameter of *ca.* 100 nm, which is in agreement with the SEM results (see Figure 1f). The nanorod exhibits a pine needle structure, which may lead to higher specific surface area than that of the nanorod with smooth or uniform surface. The TiN nanorod with high specific surface area may improve the performance in the process of H_2_O_2_ detection. Figure 3b displays the high-resolution TEM (HRTEM) image of the as-prepared TiN NRAs. The TiN crystalline grains can be seen clearly with the interplanar lattice spacing of 0.243 and 0.212 nm, corresponding well with that of (111) and (200) plane, respectively. The inset is the corresponding electron diffraction pattern, showing diffraction rings of (111) and (200) planes, which further supports the results of the XRD and HRTEM.

**Figure 6:**
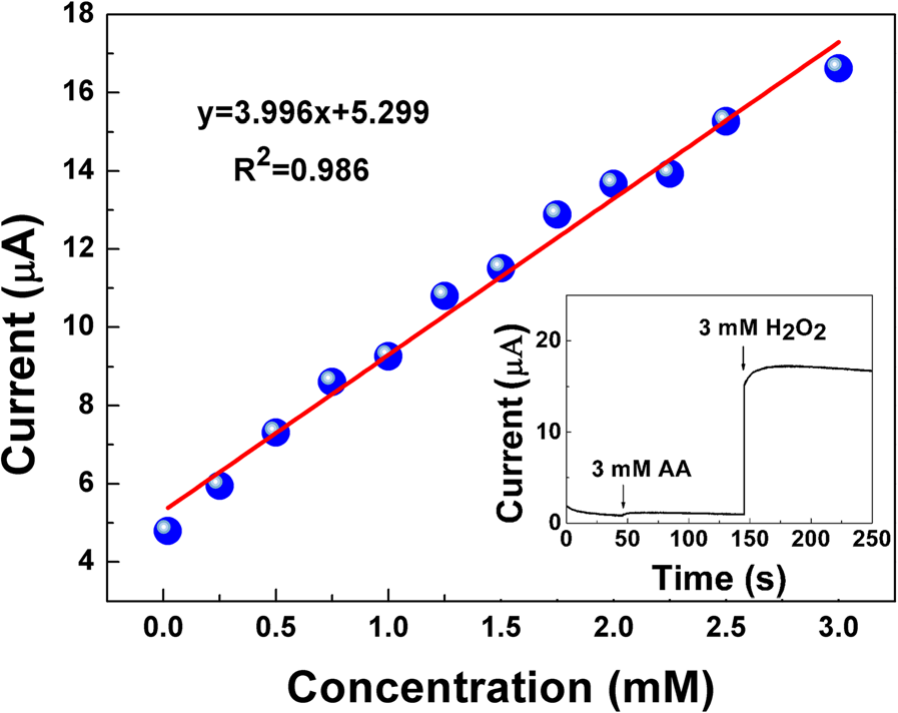
**The linear relationship between current and the concentrate of H**_**2**_**O**_**2**_**.** Inset is the current versus time after adding AA and H_2_O_2_.

The current response of TiN NRAs by successively adding different concentration H_2_O_2_ was investigated in the PBS (pH 7), and −0.2 V was selected as the applied potential. The current has a good linear relationship with the H_2_O_2_ concentration which is in the range of 2.0 × 10^−5^ to 3.0 × 10^−3^ M. The regression equation is *y* = 3.996*x* + 5.299 (*r* = 0.9930), as shown in Figure 6. Ascorbic acid (AA) is often an interference for hydrogen peroxide biosensors [20]. However, in our experiment, no interference was observed after adding 3 mM AA as shown in the inset of Figure 6. When solution of 3 mM H_2_O_2_ was added into the PBS, the reductive current increases rapidly and soon reaches stability. These results confirm that the TiN film deposited at the deposition angle of 85° possesses efficient electrocatalytic activity toward H_2_O_2_, which provides a promising way for fabricating sensors of detecting H_2_O_2_. However, compared with others' works [3,21,22], the catalytic efficiency for H_2_O_2_ of the TiN NRAs electrode is not very high. Further work is in need to improve the catalytic activity and sensitivity, such as increasing the length of TiN NRAs and enhancing the specific surface by modifying the OAD parameters.

## Conclusions

TiN films with tunable porosity were fabricated by oblique angle deposition at different deposition angles. The porosity increases with the increase of the deposition angle due to the self-shadowing effect. All the TiN films show sensitive electrochemical catalytic property towards H_2_O_2_. The film of self-standing nanorods was obtained at the deposition angle of 85° and exhibits the best performance due to its highest porosity thus the largest effective contact area with the electrolyte. Therefore, oblique angle deposition provides a promising way to fabricate TiN nanostructure as a H_2_O_2_ sensor.

## Competing interests

The authors declare that they have no competing interests.

## Authors’ contributions

ZX carried out the fabrication and characterization of the study and drafted the manuscript. XL participated in the design and coordination of the study. WW participated in the design and preparation and analyzed the results. CL participated in the design and preparation, analyzed the results, and helped draft the manuscript. ZL and ZZ participated in the design and coordination of the study. All authors read and approved the final manuscript.
